# Characteristics and survival outcomes of primary splenic cancers: A SEER population-based study

**DOI:** 10.1097/MD.0000000000028539

**Published:** 2022-01-21

**Authors:** Yanna Lei, Qian Huang, Xiaoying Li, Xiufeng Zheng, Ming Liu

**Affiliations:** Department of Abdominal Oncology, West China Hospital, Sichuan University, Chengdu, Sichuan Province, China.

**Keywords:** chemotherapy, primary splenic angiosarcoma, prognosis, SEER database, splenic lymphoma

## Abstract

Primary splenic cancers represent a small number of cancer cases and studies on its clinicopathological features and outcomes are limited. Splenic lymphomas and primary splenic angiosarcoma (PSA) are the 2 most common histological types of splenic cancers. This population-based study aimed to investigate the clinical characteristics and survival outcomes of patients with splenic lymphomas or PSA.

Patients diagnosed with splenic lymphomas or PSA between 2000 and 2015 were identified from the Surveillance Epidemiology and End Results database of the National Cancer Institutes. Overall survival (OS) and cancer-specific survival (CSS) rates were calculated using the Kaplan–Meier method. A Cox proportional hazard models were used to identify independent predictors of cancer-specific mortality.

A total of 700 patients with splenic lymphoma and 48 patients with PSA were included in this study. The median age of patients with splenic lymphoma was 65 years and 57 years for patients with PSA. For patients with splenic lymphoma, the most prevalent histological subtypes were splenic marginal zone lymphoma and diffuse large B-cell lymphoma. A total of 52.6% of the cases had stage IV disease based on the Ann Arbor staging system. Five-year OS and CSS were 76.9% and 83.4%, respectively. Multivariate analysis revealed that independent predictors of splenic lymphoma CSS included race, stage, chemotherapy, and histological subtype. However, a much shorter OS time was seen in the PSA cohort which had a 5-year OS of 11.8%, a median OS of 10.0 months and the 5-year CSS of 12.4%. Chemotherapy was correlated with better outcomes in patients with PSA. However, the survival benefits of surgery for splenic cancer were not statistically significant in our study.

The current study is the largest cohort of primary splenic cancer presented in literature based on the Surveillance Epidemiology and End Results database and our large series describe the characteristics and survival outcomes of such rare diseases which may provide reliable information for further studies and clinicians.

## 1 Introduction

The spleen is an important immune organ in our body that also has important functions in iron metabolism and erythrocyte homeostasis.^[1,2]^ Currently, there were few reports about the primary tumor, which occurs in the spleen in the clinical practice. Lymphoma is the most common primary splenic neoplasm that occurs in approximately 1% of non-Hodgkin lymphoma cases. Primary splenic angiosarcoma (PSA) is the most unusual type of splenic malignancy with a prevalence between 0.15 and 0.26 per million people.^[3–6]^

The most frequent histological subtypes of splenic lymphoma are splenic marginal zone lymphoma (SMZL) and diffuse large B-cell lymphoma (DLBCL).^[7]^ The diagnosis and classification of splenic lymphoma rely on histopathological characteristics and immunohistochemistry.^[8]^ Splenectomy, a common surgical procedure, can be used for diagnosis and is also considered a standard of care treatment for splenic lymphoma. However, the benefit of splenectomy in patients with splenic lymphoma remains controversial^[7,8]^ due to associated postoperative complications and recent advances in chemo-immunotherapy.^[9]^

PSA is a highly aggressive and extremely rare malignancy that originates from splenic vascular endothelium cells and has particularly poor survival outcomes.^[10]^ The first case of PSA was reported by Theodor Langhans in 1879 and since then only around 300 cases have been reported.^[5]^ The etiology of splenic angiosarcomas remains unclear and clinical presentations are variable. Abdominal pain is the most common symptom.^[10,11]^ The prognosis of PSA is very poor with a 20% survival rate at 6 months.^[11]^

Given the rarity of primary splenic malignant neoplasms, there is a lack of information concerning the clinicopathological characteristics of the disease and its prognosis as well as outcomes. Most available studies are case reports and small case series. Large-scale systemic analyses are not available. In this study, we outline the survival outcomes and clinical characteristics of patients with primary splenic cancers from the Surveillance Epidemiology and End Results (SEER) database of the National Cancer Institutes.

## 2 Materials and methods

### 2.1 Data source and study population

A cohort of patients diagnosed with primary splenic tumors were extracted from the SEER database. SEER database collects and publishes cancer demographics, histology, stage of the disease, treatment modalities (surgery, radiation therapy, and chemotherapy), and survival data from population-based cancer registries covering approximately 34.6% of the US population.^[12]^ By “Incidence-SEER 18 Regs Custom Data (with additional treatment fields), Nov 2018 Sub,” 748 patients were identified between 2000 and 2015. The SEER program is an open-access resource and patient information is anonymized. Therefore, this study did not require ethical review or informed consent.

The inclusion criteria for the study were as follows:

1. all patients diagnosed from 2000 to 2015,
2. the topography code was C42.2 (spleen),
3. behavior recode for analysis was malignant,
4. primary spleen cancer was the first or only cancer diagnosis,
5. histological type was confined as lymphoma (ICD-O-3 was 9590/3–9729/3) and splenic angiosarcoma (9120/3),
6. the diagnosis was pathologically confirmed. Cases with incomplete records and demographic, clinicopathological and survival data were excluded from the study.

**Figure 1:**
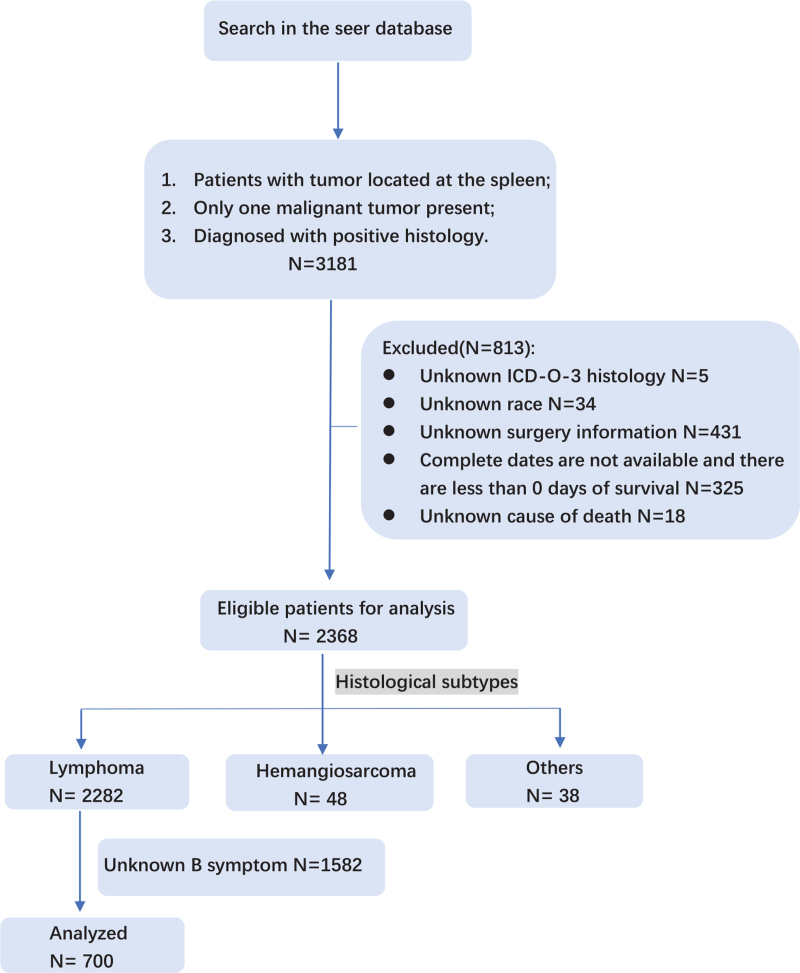
Flowchart the method for selection of the study population selection.

Initially, the study included 3181 patients who diagnosed with splenic cancer. Patients with incomplete data were removed and the remaining cases were divided into 3 groups based on pathological type. Finally, 700 patients with splenic lymphoma and 48 patients with PSA were enrolled in the study (Fig. 1). The third edition of the International Classification of Disease for Oncology is used for classify cancer histology and tomography in the SEER database. Based on the ICD-O-3 topographic codes, the histological subtypes of splenic lymphoma were divided into 3 groups; SMZL, DLBCL, and other subtypes.

### 2.2 Statistical analysis

All data were stored in Microsoft EXCEL. SEER recodes the cause of death based on International Classification of Disease 9th revision codes. OS was defined as the time from the point of diagnosis to death due to any cause. CSS was defined as the time from medical diagnosis to cancer-related death. The survival analysis was performed using an open-source R “survival” package. Deaths from cancer were considered a CSS end point and other deaths were considered censored observations. Univariable and multivariable Cox proportional hazards regression models were used to calculate hazard ratios and 95% confidence intervals. Variables that significantly affected CSS in univariable analyses were investigated by multivariate analyses. Kaplan–Meier survival curves were plotted and the differences between curves were analyzed using the log-rank test. Statistical analyses were conducted using R 4.0.3 (http://www.r-project.org) and GraphPad Prism 8. *P* < .05 was considered statistically significant.

## 3 Results

A total of 748 patients met the inclusion criteria and were included in the study. Of these patients, 700 were diagnosed with splenic lymphoma and 48 patients were diagnosed with PSA. The key demographic and clinicopathological characteristics of the study population are summarized in Table 1.

**Table 1:** Summary of the demographic and pathological characteristics of the 748 patients in this study.

Variable	Hemangiosarcoma (N = 48)	Lymphoma (N = 700)
Age		
<45	10 (20.8)	62 (8.9)
> = 45	38 (79.2)	638 (91.1)
median age	57 years	65 years
Race		
Black	1 (2.1)	51 (7.3)
Other^∗^	3 (6.2)	32 (4.6)
White	44 (91.7)	617 (88.1)
Sex		
Female	32 (66.7)	350 (50.0)
Male	16 (33.3)	350 (50.0)
B symptom		
No	–	**437 (62.4)**
Yes	–	**263 (37.6)**
Surgery		
No	10 (20.8)	376 (53.7)
Yes	38 (79.2)	324 (46.3)
Radiation		
No/Unknown	46 (95.8)	671 (95.9)
Yes	2 (4.2)	29 (4.1)
Chemotherapy		
No/Unknown	19 (39.6)	310 (44.3)
Yes	29 (60.4)	390 (55.7)
Marital status		
Married	37 (77.1)	564 (80.5)
Unknown	0 (0.0)	32 (4.6)
Unmarried	11 (22.9)	104 (14.9)
Ann Arbor Stage		
Stage I	–	223 (31.9)
Stage II	–	76 (10.9)
Stage III	–	33 (4.7)
Stage IV	–	368 (52.6)

### 3.1 Baseline characteristics and treatment modalities of splenic lymphomas

The median age of the patients in the splenic lymphoma group was 65 years (range, 25–90) of which 88.1% were of White race. The proportion-of males and females was 1:1 for the incidence of splenic lymphoma and 52.6% presented with stage IV disease. The median follow-up time was approximately 32 months and 82% of patients were alive at the end of the follow-up. Lymphoma still accounted for the most cases of deaths. Table 2 summarizes the distribution of all histological subtypes in the splenic lymphoma cohort. The most prevalent tumor histological subtype was SMZL (50.3%) followed by DLBCL (27.3%). The proportion of patients with B symptoms (37.6%) was lower than those who did not have B symptoms (62.4%). In the subgroup analysis stratified by histological subtypes (Table 3), DLBCL patients were more likely to receive surgery as well as chemotherapy-, had early-stage disease and presented with B symptoms compared to patients in the SMZL subgroup.

**Table 2:** Histological subtypes of primary lymphoma of splenic tumors.

Histology	N
Splenic marginal zone lymphoma	352
Diffuse large B-cell lymphoma	191
Non-Hodgkin lymphoma, NOS	49
Hepatosplenic T-cell lymphoma	34
Follicular lymphoma	31
Mantle cell lymphoma	18
Malignant lymphoma, NOS	8
Peripheral T-cell lymphoma, NOS	6
Hodgkin lymphoma	4
Lymphoplasmacytic lymphoma	3
Anaplastic large cell lymphoma, ALK-positive	2
Extranodal NK-/T-cell lymphoma, nasal type	1
Primary cutaneous gamma-delta T-cell lymphoma	1

**Table 3:** Summary of the clinical characteristics of patients with primary splenic lymphoma in the SEER database (2010–2015) stratified by histology.

	DLBCL	SMZL	Others	Overall
	(N = 191)	(N = 352)	(N = 157)	(N = 700)
Age				
<45	16 (8.4%)	20 (5.7%)	26 (16.6%)	62 (8.9%)
> = 45	175 (91.6%)	332 (94.3%)	131 (83.4%)	638 (91.1%)
Race				
Black	12 (6.3%)	24 (6.8%)	15 (9.6%)	51 (7.3%)
Other	8 (4.2%)	13 (3.7%)	11 (7.0%)	32 (4.6%)
White	171 (89.5%)	315 (89.5%)	131 (83.4%)	617 (88.1%)
Sex				
Female	100 (52.4%)	182 (51.7%)	68 (43.3%)	350 (50.0%)
Male	91 (47.6%)	170 (48.3%)	89 (56.7%)	350 (50.0%)
B symptom				
No	107 (56.0%)	238 (67.6%)	92 (58.6%)	437 (62.4%)
Yes	84 (44.0%)	114 (32.4%)	65 (41.4%)	263 (37.6%)
Surgery				
No	67 (35.1%)	214 (60.8%)	95 (60.5%)	376 (53.7%)
Yes	124 (64.9%)	138 (39.2%)	62 (39.5%)	324 (46.3%)
Radiation				
No/Unknown	173 (90.6%)	350 (99.4%)	148 (94.3%)	671 (95.9%)
Yes	18 (9.4%)	2 (0.6%)	9 (5.7%)	29 (4.1%)
Chemotherapy				
No/Unknown	33 (17.3%)	220 (62.5%)	57 (36.3%)	310 (44.3%)
Yes	158 (82.7%)	132 (37.5%)	100 (63.7%)	390 (55.7%)
Stage				
Stage I	99 (51.8%)	75 (21.3%)	49 (31.2%)	223 (31.9%)
Stage II	39 (20.4%)	23 (6.5%)	14 (8.9%)	76 (10.9%)
Stage III	15 (7.9%)	8 (2.3%)	10 (6.4%)	33 (4.7%)
Stage IV	38 (19.9%)	246 (69.9%)	84 (53.5%)	368 (52.6%)

Surgery was more frequently performed for patients with early-stage disease, however, 34.3% of patients with stage IV disease also received surgery. Overall, 46.3% of patients underwent surgery and 55.7% underwent chemotherapy. The proportion of patients receiving radiation therapy was 4.2%. The proportion of patients treated with surgery was the lowest in the SMZL group (39.2%) and highest in the DLBCL group (64.9%). A total of 154 patients were treated with both surgery and chemotherapy and 140 patients underwent no treatment in this cohort.

### 3.2 Baseline characteristics and treatment modalities of splenic angiosarcoma

Detailed information on the 48 patients with PSA was extracted from the SEER database. The median age of all patients was 57years and the median follow-up was 8 months. A total of 79.2% of patients were aged more than 45 years old and more than half of the patients were female. Most of the patients were White (91.7%) and most patients were married (77.1%). Among these patients, 79.2% were treated with surgery and 60.4% were treated with chemotherapy. Nine patients (18.8%) did not undergo surgery or chemotherapy.

### 3.3 Survival outcomes of splenic lymphomas

**Figure 2:**
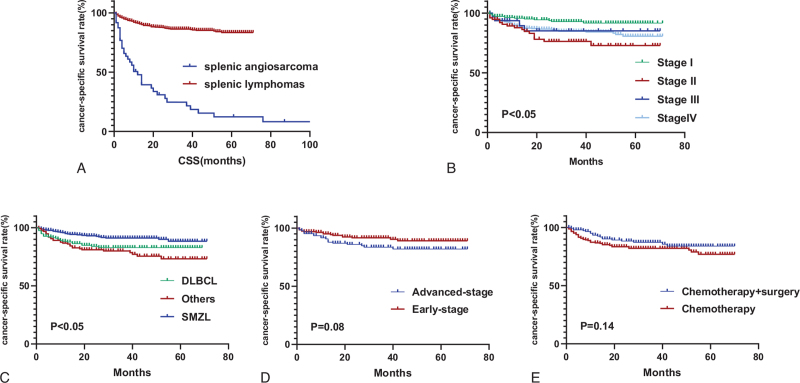
Survival analysis of the SEER Cohort. (A) Kaplan–Meier curves for CSS of patients with splenic lymphoma and PSA; (B, C) Kaplan–Meier survival curves for CSS stratified by stage and histology in patients with splenic lymphoma; (D) Kaplan–Meier survival curves for early or advanced-stage patients with splenic lymphoma who underwent surgery; (E) Kaplan–Meier survival curves for patients with splenic lymphoma who received chemotherapy or combination therapy.

The 5-year CSS and OS rates of patients with splenic lymphoma were 83.4% and 76.9%, respectively (Fig. 2A). Univariate analysis showed that race, histology, stage, and chemotherapy were closely related to CSS (all *P* < .05, Table 4). Other clinical factors including gender, age, surgery, B symptoms, marital status and radiotherapy were not correlated with survival (*P* > .05). According to stratification by stage of the disease, 5-year CSS estimates were 91.6% for patients with stage I, 72.8% for stage II, 85.1% for stage III, and 80.7% for stage IV (Fig. 2B). CSS was found to be significantly different between patients with stage I and II disease as well as stage I and IV.

**Table 4:** Cox proportional hazards regression model for CSS in splenic lymphoma (n = 700).

	Variables univariate analysis		Multivariate analysis	
	HR [95% CI]	*P*	HR [95% CI]	*P*
Age				
<45	Ref			
> = 45	0.61[0.33,1.12]			
Race				
Black				
Other	1.14[0.46,2.846]	.77	1.07 [0.43, 2.68]	.89
White	0.46[0.25,0.88]	.0172	0.49 [0.25, 0.93]	.03
Sex				
Female	Ref			
Male	1.40[0.886,2.07]	.17		
Histology				
DLBCL	Ref			
SMZL	0.41[0.29,0.83]	.008	0.37 [0.19, 0.70]	.002
Others	1.32[0.80,2.21]	.28	1.11 [0.63, 1.96]	.72
B symptom				
No	Ref			
Yes	1.41[0.92,2.15]	.11		
Stage				
Stage I	Ref			
tage II	3.53[1.74,7.17]	.0005	3.47 [1.70, 7.06]	.001
tage III	2.09[0.69,6.34]	.19	1.89 [0.62, 5.78]	.27
Stage IV	2.25[1.25,4.07]	.0071	3.02 [1.59, 5.72]	.001
Marital status				
Married	Ref			
Unknown	0.25[0.04,1.83]	.17		
Unmarried	1.43[0.84,2.45]	.18		
Surgery				
No	Ref			
Yes	0.73[0.48,1.13]	.16		
Radiation				
No/Unknown	Ref			
Yes	0.82[0.26,2.60]	.74		
Chemotherapy				
No/Unknown	Ref			
Yes	1.6[1.02,2.52]	.0401	1.05 [0.64, 1.73]	.84

The 5-year CSS rate was 82.9% for patients with DLBCL, 88.3% for patients with SMZL and 73.2% for other histological subtypes (Fig. 2C). Patients who had early-stage disease, without B symptoms and with SMZL were more likely to receive surgery, although surgery did not significantly affect CSS. The 5-year CSS rate was 89.1% for early-stage patients who received surgery and 82% for advanced-stage patients who underwent surgery (Fig. 2D, *P* = .08). A total of 154 patients received chemotherapy and surgery. Our results showed that surgery combined with chemotherapy did not improve patient outcomes compared to chemotherapy alone. The 5-year CSS rates in patients received chemotherapy combined with surgery and chemotherapy alone were 84.0% and 76.9%, respectively (*P* = .14, Fig. 2E). Race, histology, and stage were affecting CSS in the multivariate analyses.

### 3.4 Survival outcomes of primary splenic angiosarcoma

**Figure 3:**
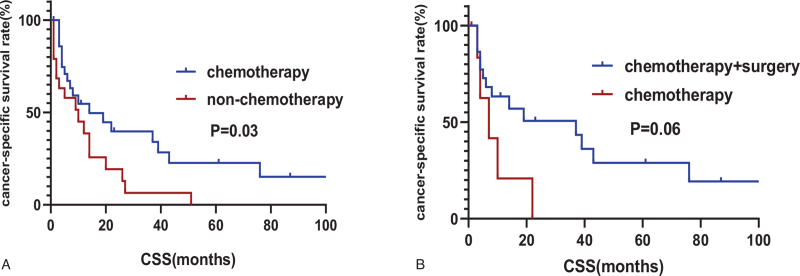
Survival analysis of the SEER Cohort (A) Kaplan–Meier survival curves for received chemotherapy or non-chemotherapy patients with PSA; (B) Kaplan–Meier survival curves for patients with PSA who received chemotherapy or combination therapy.

Compared to patients with splenic lymphoma, PSA patients have a particularly poor prognosis. The median survival time was 10 months. The 1-year CSS and OS were 47.8% and 45.8%, respectively. The 5-year OS was 11.8% and the 5-year CSS was 12.4% (Fig. 2A). The only factor that was significantly associated with improved CSS on univariate analysis was chemotherapy (*P* = .037, Table 5). Multivariate analysis was performed with variables including the treatment modalities such as surgery, radiation therapy, as well as chemotherapy and revealed that chemotherapy was independent prognostic factor for PSA patients. The median survival time in patients treated with chemotherapy was 14 months and for patients that were not treated with chemotherapy the median survival time was 10 months (*P* = .03, Fig. 3A). Surgery and radiotherapy were not significantly related to the prognosis of PSA patients.

**Table 5:** Cox proportional hazards regression model for CSS in PSA (n = 48).

	Variables univariate analysis		Multivariate analysis	
	HR [95% CI]	*P*	HR [95% CI]	*P*
Age				
<45	Ref			
> = 45	0.18 [0.77,4.04]	.18		
Sex				
Female	Ref			
Male	0.89[0.43,1.87]	.76		
Marital status				
Married	Ref			
Unmarried	0.88 [0.39,2.02]	.77		
Surgery				
No	Ref		Ref	
Yes	0.49 [0.21,1.18]	.11	0.43[0.17,1.04]	.06
Radiation				
No/Unknown	Ref		Ref	
Yes	0.39[0.05,2.92]	.36	0.49[0.06,3.77]	.49
Chemotherapy				
No/Unknown	Ref		Ref	
Yes	0.49 [0.25,0.96]	.037	0.46[0.23,0.92]	.028

In the study cohort, 29 patients underwent surgery of which 22 received chemotherapy and surgery, and 7 patients received surgery alone. However, surgery combined with chemotherapy did not improve the CSS of PSA compared to treatment with chemotherapy alone (*P *= .06, Fig. 3B).

## 4 Discussion

Due to the rarity of splenic cancers, data on this disease are still lacking. In this study, we analyzed the clinical characteristics and survival outcomes of 2 common types of splenic tumors. To the best of our knowledge, this is the first study to explore the clinical features of primary splenic cancers based on data from the SEER database. Our study provides relevant information for clinicians that may facilitate more appropriate management strategies in the clinic. This study also identified the effects of conventional treatments such as surgery and chemotherapy on splenic lymphoma and PSA.

Splenic lymphoma refers to tumors confined to the spleen and the hilar lymph nodes,^[13]^ which can be further classified as SMZL, DLBCL, and other subtypes such as follicular lymphoma, according to its pathological type. In this study, SMZL was the most common type of splenic lymphoma which was in contrast to previous findings in a study of 115 patients that showed DLBCL was the most common subtype followed by SMZL.^[14]^ The median age of patients in this study was 66.0 years old. Another study revealed the median age was 55.4 years old and the male to female ratio was 3.4:1.^[15]^ However, in our cohort, the ratio male to female ratio was 1:1. The discrepancy may due to the limited sample size in the former study that included only 53 cases while 700 patients were included in our analysis. This study also confirmed the SMZL was the most common subtype, not the DLBCL. And the incidence of B symptoms in this small-scale research was 35.4% and 41.4% in our article.

Multivariate analysis revealed several independent risk factors that affect the prognosis of splenic lymphoma. Non-White race and advanced stage were associated with poor prognosis. Many studies have found that race could serve as a prognostic factor for various cancers such as hepatocellular carcinoma,^[16]^ lymphoma,^[17–19]^ and breast cancer.^[20]^ Lower treatment rates and delayed onset of treatment may account for this phenomenon. The pathological type of disease is also a key factor of prognosis. Our study showed that patients with SMZL have a better prognosis which was consistent with previous studies.^[14,15]^ In addition, it was reported that DLBCL had a higher age, higher lactate dehydrogenase and tumor formation in the spleen compared with SMZL.^[14]^ The stage of disease is an independent prognostic factor and patients with advanced-stage disease are more likely to have poor outcomes. However, there was no statistical significance between patients with stage I and stage III disease which may be due to the low numbers of patients with stage III disease. In addition, Ahmann et al grouped malignant lymphoma of the spleen into 3 stages.

Stage 1: the tumor was limited to the spleen;
Stage 2: the nodes in the splenic hilum were involved as well;
Stage 3: the involvement of the liver or lymph nodes beyond the splenic hilum.^[21]^

In our study, the staging of splenic lymphoma was based on Ann Arbor stage. The stage of PSA was unclear and we cannot find the stage information in medical record.

The optimal therapeutic strategy is yet to be defined for patients with splenic cancer. Splenectomy has been previously recommended for patients^[7,10,15,22]^ as it can relieve the adverse effects on blood counts and contribute to disease control. However, recent findings indicate that splenectomy may not be the best choice for treatment due to immune suppression and the increased risk of infection after surgery.^[22]^ Diagnosis may rely on other methods of detection such as analysis of the blood and bone marrow.^[7]^ In our study, no significant survival benefit from surgery was found. Our data also demonstrated the effectiveness of chemotherapy, yet combined splenectomy and chemotherapy did not have a significant influence on survival outcomes compared to chemotherapy alone (*P* > .05).

PSA is characterized by low incidence, poor prognosis and does not have specific clinical signs.^[23,24]^ It was reported that PSA has high rates of regional and distant metastases and local recurrence.^[25]^ Tumors are more frequent in middle-aged and older patients.^[5,25]^ In our study, the median age of PSA patients was 57 years and the male to female ratio was 1:2. The pathogenesis of PSA remains unknown and pain in the left upper abdomen is the common clinical presentation.^[10]^ A retrospective study in China aimed to investigate the clinical characteristics and prognosis of PSA.^[5]^ This study showed the rate of splenic rupture was 59.11% in PSA and splenic rupture before surgery, tumor size as well as adjuvant chemotherapy were associated with survival outcomes. Patients with the splenic rupture before surgery or large tumors (>5 cm) are more likely to have worse outcomes.

In our study, chemotherapy was significant correlated with PSA patients. Chemotherapy significantly improved the prognosis of PSA but combined splenectomy and chemotherapy did not show a significant survival advantage compared to chemotherapy alone. Also, the role of surgery in PSA remains unclear. Previous studies have shown a survival advantage for patients undergoing surgery before splenic rupture.^[10,26]^ However, in our study, surgery was not correlate with survival.

Our study also has several limitations. First, the data were obtained from the SEER database which imparts an inherent bias in the retrospective analysis. Second, several important variables that may impact affect prognosis are not included in this study. For example, it was reported that splenic cancer may have different clinical manifestations but these were simply reported as abdominal discomfort or spleen enlargement without any other abnormalities.^[10,27]^ Third, abnormal blood counts neutropenia, decreased hemoglobin, thrombocytopenia, or lymphocytosis can also be found through laboratory examinations.^[15]^ Elevated lactate dehydrogenase levels also can be seen in patients with splenic lymphoma that also correlate with a worse prognosis.^[15]^ Finally, specific chemotherapy regimens are not available in the SEER database, so we still cannot provide a proper treatment plan for patients. Additionally, the role of radiotherapy was not discussed in our study due to the limited number of patients in the SEER database.

In conclusion, our study used the largest database to gain valuable information about the clinical characteristic of splenic cancer patients that may inform decision-making in the clinic. This study identifies the factors that are correlated with CSS and highlights the impact- of surgery and chemotherapy on the survival of patients with splenic cancer. Further validation of these findings is required to determine the optimum management strategies for patients with rare splenic tumors.

## Author contributions

**Conceptualization:** Ming Liu.

**Formal analysis:** Yanna Lei.

**Supervision:** Qian Huang, Ming Liu.

**Validation:** Xiaoying Li, Xiufeng Zheng, Ming Liu.

**Writing – review & editing:** Yanna Lei.
